# Per-COVID-19: A Benchmark Dataset for COVID-19 Percentage Estimation from CT-Scans

**DOI:** 10.3390/jimaging7090189

**Published:** 2021-09-18

**Authors:** Fares Bougourzi, Cosimo Distante, Abdelkrim Ouafi, Fadi Dornaika, Abdenour Hadid, Abdelmalik Taleb-Ahmed

**Affiliations:** 1 Institute of Applied Sciences and Intelligent Systems, National Research Council of Italy, 73100 Lecce, Italy; fares.bougourzi@isasi.cnr.it; 2 Laboratory of LESIA, University of Biskra, Biskra 7000, Algeria; ou_karim@yahoo.fr; 3 University of the Basque Country UPV/EHU, 20018 San Sebastian, Spain; fadi.dornaika@ehu.eus; 4 IKERBASQUE, Basque Foundation for Science, 48009 Bilbao, Spain; 5 University Polytechnique Hauts-de-France, University Lille, CNRS, Centrale Lille, UMR 8520-IEMN, F-59313 Valenciennes, France; abdenour.hadid@ieee.org (A.H.); Abdelmalik.Taleb-Ahmed@uphf.fr (A.T.-A.)

**Keywords:** COVID-19, deep learning, convolutional neural network, CT-scans, dataset generation

## Abstract

COVID-19 infection recognition is a very important step in the fight against the COVID-19 pandemic. In fact, many methods have been used to recognize COVID-19 infection including Reverse Transcription Polymerase Chain Reaction (RT-PCR), X-ray scan, and Computed Tomography scan (CT- scan). In addition to the recognition of the COVID-19 infection, CT scans can provide more important information about the evolution of this disease and its severity. With the extensive number of COVID-19 infections, estimating the COVID-19 percentage can help the intensive care to free up the resuscitation beds for the critical cases and follow other protocol for less severity cases. In this paper, we introduce COVID-19 percentage estimation dataset from CT-scans, where the labeling process was accomplished by two expert radiologists. Moreover, we evaluate the performance of three Convolutional Neural Network (CNN) architectures: ResneXt-50, Densenet-161, and Inception-v3. For the three CNN architectures, we use two loss functions: MSE and Dynamic Huber. In addition, two pretrained scenarios are investigated (ImageNet pretrained models and pretrained models using X-ray data). The evaluated approaches achieved promising results on the estimation of COVID-19 infection. Inception-v3 using Dynamic Huber loss function and pretrained models using X-ray data achieved the best performance for slice-level results: 0.9365, 5.10, and 9.25 for Pearson Correlation coefficient (PC), Mean Absolute Error (MAE), and Root Mean Square Error (RMSE), respectively. On the other hand, the same approach achieved 0.9603, 4.01, and 6.79 for $PC_{subj}$, $MAE_{subj}$, and $RMSE_{subj}$, respectively, for subject-level results. These results prove that using CNN architectures can provide accurate and fast solution to estimate the COVID-19 infection percentage for monitoring the evolution of the patient state.

## 1. Introduction

Since the end of 2019, the World has faced a health crisis because of the COVID-19 pandemic. The crisis has influenced many aspects of human life. To save the infected persons’ lives and stop the spread of COVID-19, many methods have been used to recognize the infected persons. These methods include Reverse Transcription Polymerase Chain Reaction (RT-PCR) [1], X-ray scan [2,3,4], and CT-scan [5,6]. Despite the fact that the RT-PCR test is considered as the global standard method for COVID-19 diagnosis, this method has many downsides [7,8]. In detail, the RT-PCR test is time-consuming, expensive, and has a considerable False-Negative Rate [1]. Using X-ray scan and CT-scan methods can replace RT-PCR test and give an efficient result in both time and accuracy [2,8]. However, both of these methods need an expert radiologist to identify COVID-19 infection. Artificial Intelligence (AI) can provide the right solution to make this process automatic and limit the need of the radiologist to recognize the COVID-19 infection from these medical imaging. Indeed, computer vision and machine learning communities have proposed many algorithms and frameworks which have proved their efficiency on this task. Especially by using deep leaning methods which already have proved their efficiency on different computer vision tasks [9] including medical imaging tasks [10,11].

Compared with the other two aforementioned diagnosis methods, the CT scan method has many advantages, as shown in Table 1. In addition to the use of CT scans to recognize COVID-19 infection, they can be used for other important tasks, which include quantifying the infection and monitoring the evolution of the disease, which can help with treatment and save the patient’s life [12]. Moreover, the evolution stage can be recognized, where the typical signs of COVID-19 infection could be ground-glass opacity (GGO) in the early stage, and pulmonary consolidation in the late stage [7,8]. According to the estimated COVID-19 infection percentage from the CT-scans, the patient state can be classified into Normal (0%), Minimal (<10%), Moderate (10–25%), Extent (25–50%), Severe (50–75%), and Critical (>75%) [13].

Most of the state-of-the art methods have been concentrating on the recognition of COVID-19 from the CT scans or segmentation of the infected regions. Despite the huge efforts that have been made, the state-of-the-art methods have not provided many helpful tools to monitor the patient state, the evolution of the infection, or the response of patient to the treatment, which can play a crucial role in saving the patient’s life. In this paper, we propose a fully automatic approach to evaluate the evolution of COVID-19 infection from the CT scans as regression task which can provide a richer information about the COVID-19 infection evolution. The estimation of COVID-19 percentage can help intensive care workers to identify the patients that need urgent care, especially the critical and severe cases. With the extensive number of COVID-19 infections, estimating the COVID-19 percentage can help intensive care workers free up resuscitation beds for the critical cases and follow other protocol for less severe cases.

Unlike the mainstream that dealt with COVID-19 recognition and segmentation, this paper addresses the estimation of COVID-19 infection percentage. To this end, we constructed the Per-COVID-19 dataset, then we used it to evaluate the performance of three CNN architectures with two loss functions and two pretrained models scenarios. In summary, the main contributions of this paper are as follows:

- We introduce the Per-COVID-19 dataset for estimating the COVID-19 infection percentage for both slice-level and patient-level. The constructed dataset consists of 183 CT scans with the corresponding slice-level COVID-19 infection percentage which were estimated by two expert radiologists. To the best of our knowledge, our work is the first to propose a finer granularity of COVID-19 virus presence and solve a challenging task related to exact estimation of COVID-19 infection percentage.
- In order to test some state-of-the art methods, we evaluated the performance of three CNN architectures: ResneXt-50, Densenet-161, and Inception-v3. For the three CNN architectures, we use two loss functions: Mean Squared Error (MSE) and Dynamic Huber loss. In addition, two pretrained scenarios are investigated. In the first scenario, the pretrained models on ImageNet are used. To study the influence of using pretrained models on medical imaging task, we use the pretrained models on X-ray images.
- We make our database and codes publicly available to encourage other researchers to use it as a benchmark for their studies https://github.com/faresbougourzi/Per-COVID-19 (last accessed on 4 August 2021).

## 2. Related Works

The state-of-the-Art methods using CT scans can be classified into two main tasks: COVID-19 recognition [5,6,14,15,16] and COVID-19 segmentation [7,8,17,18]. In [19], Zheng, C. et al. proposed the DeCoVNet approach, which is based on 3D deep convolutional neural Network to Detect COVID-19 (DeCoVNet) from CT volumes. The input to DeCoVNet is CT volume, and its 3D lung mask was generated by using pretrained UNet [20]. Their proposed DeCoVNet architecture has three parts: vanilla 3D convolution, 3D residual blocks (ResBlocks), and progressive classifier (ProClf). He, K. et al. proposed a multi-task multi-instance deep network (M${}^{2}$UNet) to assess the severity of COVID-19 patients [21]. Their proposed approach classifies the volumetric CT-scans into two classes of severity: severe or non-severe. Their M${}^{2}$UNet approach consists of a patch-level encoder, a segmentation subnetwork for lung lobe segmentation, and a classification subnetwork for severity assessment. In [22], Yao, Q. et al. proposed the NormNet architecture, which is a voxel-level anomaly modeling network, to distinguish healthy tissues from the COVID-19 lesion in the thorax area. Paulo. L. et al. investigated transfer learning and hyperparameter optimization techniques to improve the computer-aided diagnosis for COVID-19 recognition sensitivity [15]. To this end, they tested different data preprocessing and augmentation techniques. In addition, four CNN architectures were used and four hyperparameters were optimized for each CNN architecture.

Zhao, X.et al. proposed a dilated dual-attention U-Net (D2A U-Net) approach for COVID-19 lesion segmentation in CT slices based on dilated convolution and a novel dual-attention mechanism to address the issues above [7]. In [17], Alessandro. S. et al. proposed a customized ENET (C-ENET) approach for COVID-19 infection segmentation. Their proposed C-ENET approach proved its efficiency in public datasets compared with UNET [20] and ERFNET [23] segmentation architectures. To deal with the limitation of the training data for segmenting COVID-19 infection, Athanasios. V. et al. introduced the few-shot learning (FSL) concept of network model training using a very small number of samples [18]. They explored the efficiency of few-shot learning in U-Net architectures, allowing for a dynamic fine-tuning of the network weights as new few samples are being fed into the UNet. Experimental results indicate improvement in the segmentation accuracy of identifying COVID-19 infected regions.

The main limitation of the state-of-the-art works is that they have been concentrating on the recognition and segmentation of COVID-19 infection. However, more information about the disease evolution and severity could be inferred from the CT scans. On the other hand, the available datasets are very limited, especially for the segmentation and severity tasks. Table 2 shows some of the available Segmentation datasets. From this table, we notice that these datasets were contrasted with a small number of CT scans and slices. This is because the time and effort required for the labeling process is very large, with most radiologists having very little time, especially during this pandemic. In this work, we have created a dataset for estimating the percentage of COVID-19 infections that requires less time and effort for the labeling process.

## 3. Materials and Methods

### 3.1. Per-COVID-19 Dataset

**Figure 1:**
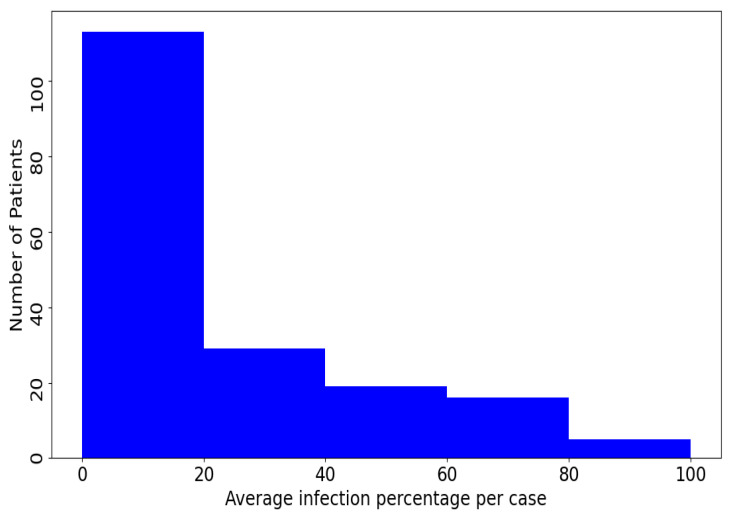
Histogram of the slices average infection percentage per case in the proposed Per-COVID-19 database.

Our Per-COVID-19 database consists of 183 CT scans that were confirmed to have COVID-19 infection. Figure 1 shows the histogram of the infection percentage per case. The patients were from both genders and aged between 27 to 70 years old. Each volumetric CT scan of the Per-COVID-19 database was taken from a different patient, so the number of CT scans equals to the number of patients. The diagnosis of COVID-19 infection is based on positive reverse transcription polymerase chain reaction (RT-PCR) and CT scan manifestations identified by two experienced thoracic radiologists. The CT scans were collected from two hospitals: Hakim Saidane Biskra and Ziouch Mohamed Tolga (Algeria) from June to December 2020. Each CT scan consists of 40–70 slices. Table 3 summarizes the number of CT-scans and the used recording settings and device for each hospital. The two Radiologists estimated COVID-19 infection percentage based on the area of infected lungs over the overall size of the lungs. From each CT scan, the radiologists picked the slices that contain signs of COVID-19 infection and the ones that do not contain any COVID-19 signs.

**Figure 2:**
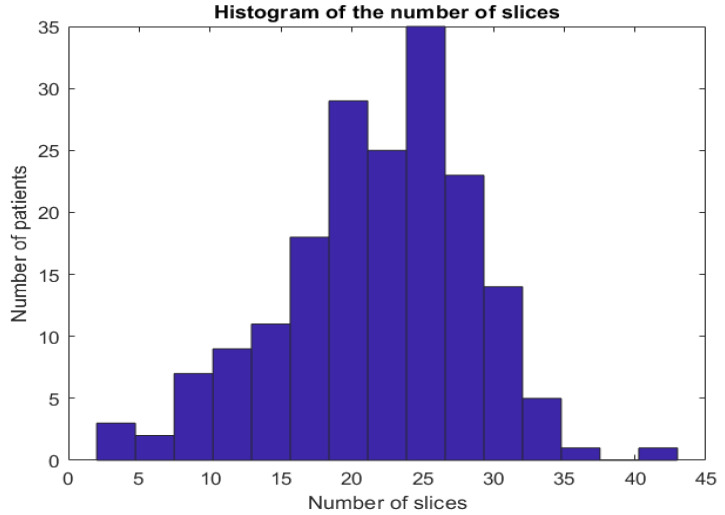
Histogram of slices number per patient in the proposed Per-COVID-19 database.

**Figure 3:**
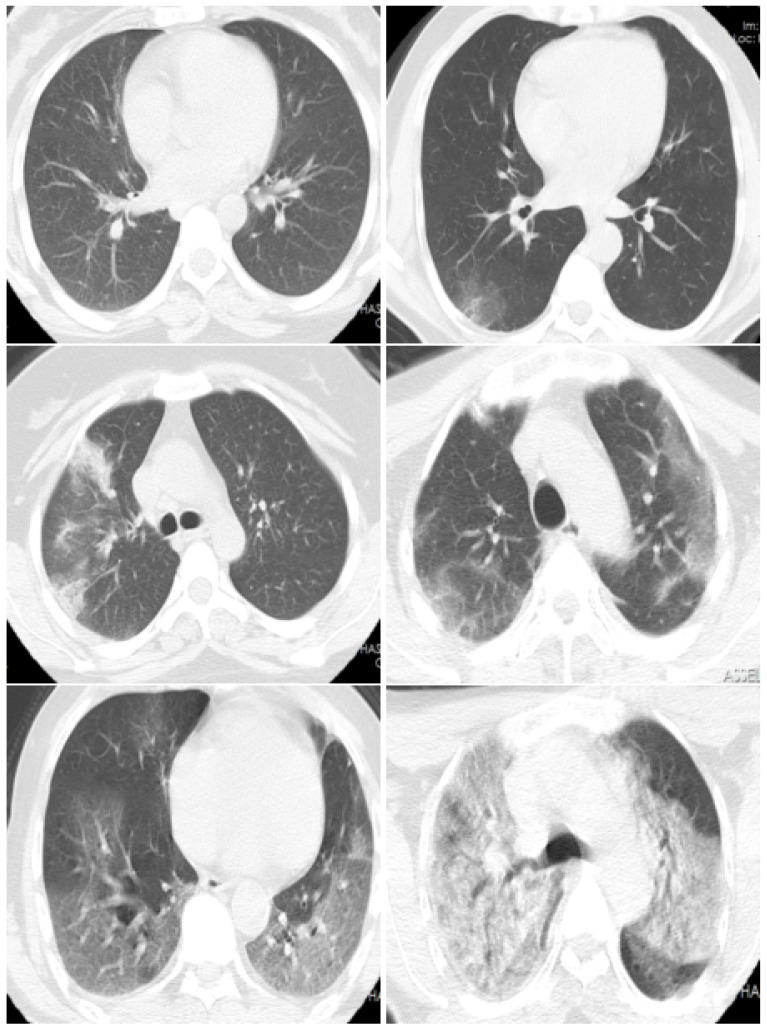
CT scan images examples which have the COVID-19 infection percentages of 0%, 5%, 18%, 40%, 70%, and 80%, respectively. These slices are classified as Normal, Minimal (<10%), Moderate (10–25%), Extent (25–50%), Severe (50–75%), and Critical (>75%), respectively.

In the proposed dataset, we kept the slices that have diagnosis agreement of the two radiologists. In summary, from each CT-scan, we can have between 2 and 45 typical slice images (these slices have close estimation of COVID-19 infection percentage from both radiologists). Figure 2 shows the histogram of number of CT-scans over the number of CT slices. Figure 3 shows examples of slices images with their corresponding COVID-19 infection percentage.

**Figure 4:**
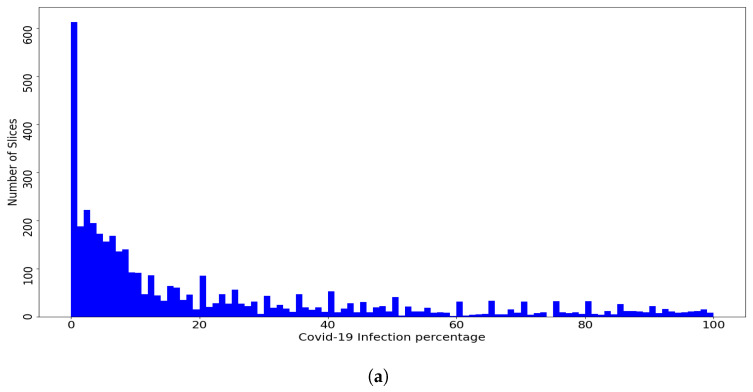

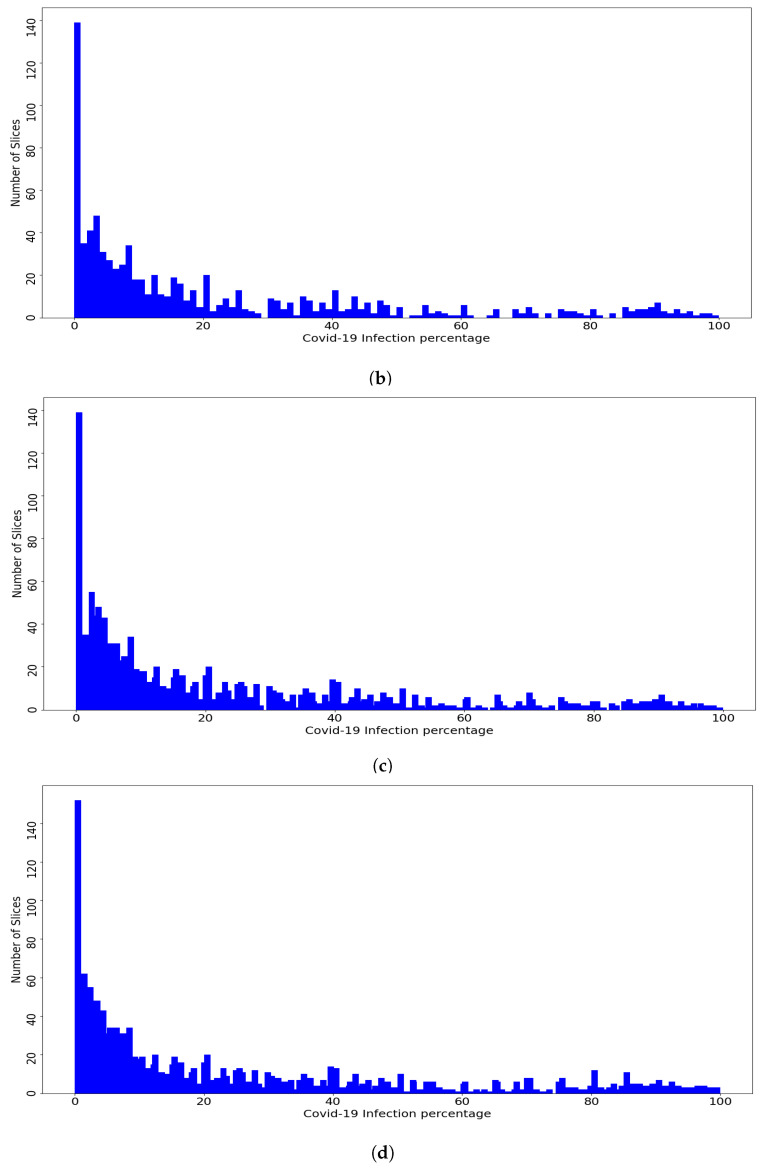

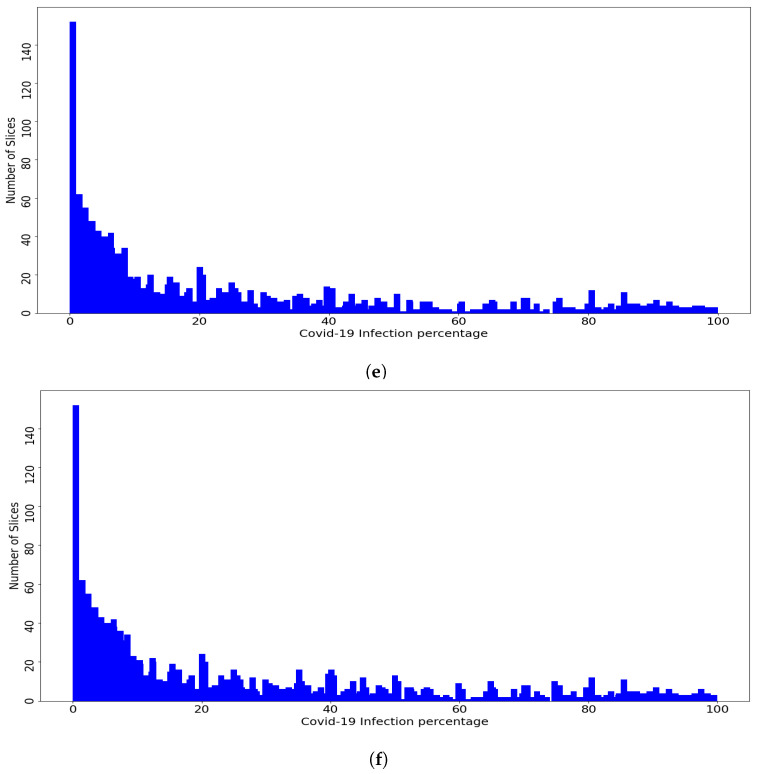
(**a**) Per-COVID-19 Dataset, (**b**) Fold1. (**c**) Fold2, (**d**) Fold3. (**e**) Fold4, and (**f**) Fold5. Histogram of COVID-19 infection percentage per slice for the dataset and the five folds.

To evaluate different machine learning methods, we divided the database Per-COVID-19 into five patient-independent folds, with each patient slice contained in only one fold. Figure 4 shows the distribution of the slices of the dataset and the fold slices over the percentage of COVID-19 infections, where the dataset and the fold slices have an almost similar distribution. The goal of using the Patient-Independent evaluation protocol is to test the performance of the CNN architectures for slices from new patients not seen in the training phase. In total, there are 3986 labeled slices with the corresponding COVID-19 infection percentage. These slices were converted to PNG format and the lung regions were then manually cropped. The dataset is available at https://github.com/faresbougourzi/Per-Covid-19 (accessed on 4 August 2021), with labels available in the file ‘Database_new.xlsx’. The columns represent the image name, percentage of COVID-19 infection, fold split number, and subject ID.

### 3.2. Methods

#### Loss Functions

In our experiments, we used two loss functions: Mean Squared Error (MSE) and Dynamic Huber loss. The loss functions are defined for *N* batch size, and $X=(x_{1},x_{2},\ldots ,x_{N})$ are the ground-truth percentages and $\hat{X}=\left(\hat{x_{1}},\hat{x_{2}},\ldots ,\hat{x_{N}}\right)$ are their corresponding estimated percentages.

MSE is sensitive towards outliers. For *N* predictions, MSE loss function is defined by

(1) $$L_{MSE}=\frac{1}{N}\sum_{i=1}^{N}{\left(x_{i}-\hat{x_{i}}\right)}^{2}$$

on the other hand, the Huber loss function is less sensitive to outliers in data than $L_{2}$ loss function. For *N* training batch size images, the Huber loss function is defined by [27]:

(2) $$L_{Huber}=\frac{1}{N}\sum_{i=1}^{N}z_{i}$$

where *N* is the batch size and $z_{i}$ is defined by

(3) $$z_{i}=\left\{\begin{array}{cc} 0.5\;{\left(x_{i}-\hat{x_{i}}\right)}^{2}, & if\;|x_{i}-\hat{x_{i}}|\leq \beta \\ \beta \;|x_{i}-\hat{x_{i}}|-0.5\;\beta^{2}, & \mathrm{otherwise} \end{array}\right.$$

where β is a controlling hyperparameter. In our experiments, β decreases from 15 to 1 during the training.

### 3.3. Evaluation Metrics

To evaluate the performance of the state-of-the-art methods, we use slice-level metrics which are Mean Absolute Error (MAE), Root Mean Square Error (RMSE), and Pearson Correlation coefficient (PC), which are defined in Equations (4)–(6), respectively.

(4) $$MAE=\frac{1}{n}\sum_{i=1}^{n}|y_{i}-\hat{y_{i}}|$$

(5) $$RMSE=\frac{1}{n}\sum_{i=1}^{n}{\left(y_{i}-\hat{y_{i}}\right)}^{2}$$

(6) PC=∑i=1n(yi−yi¯)(yi^−yi^¯)(∑i=1n(yi−yi¯)2∑i=1n(yi^−yi^¯)2

where $Y=(y_{1},y_{2},\ldots ,y_{n})$ are the ground-truth COVID-19 percentages of the testing data which consists of *n* slices and $\hat{Y}=\left(\hat{y_{1}},\hat{y_{2}},\ldots ,\hat{y_{n}}\right)$ are their corresponding estimated percentages. For Equation (6), $\bar{y_{i}}$ and $\bar{\hat{y_{i}}}$ are the means of the ground-truth percentages and the estimated ones, respectively.

In addition, we used subject-level metrics $MAE_{subj}$, $RMSE_{subj}$, and $PC_{subj}$, which are defined in Equations (7)–(9), respectively.

(7) $$MAE_{subj}=\frac{1}{s}\sum_{i=1}^{s}|y_{s_{i}}-\hat{y_{s_{i}}}|$$

(8) $$RMSE_{subj}=\frac{1}{s}\sum_{i=1}^{s}{\left(y_{s_{i}}-\hat{y_{s_{i}}}\right)}^{2}$$

(9) PCsubj=∑i=1s(ysi−ysi¯)(ysi^−ysi^¯)(∑i=1s(ysi−ysi¯)2∑i=1s(ysi^−ysi^¯)2

where $Y_{s}=\left(y_{s_{1}},y_{s_{2}},...,y_{s_{n}}\right)$ are the ground-truth means of COVID-19 percentages of each patient’ slices from the testing data and $\hat{Y_{s}}=\left(\hat{y_{s_{1}}},\hat{y_{s_{2}}},...,\hat{y_{s_{s}}}\right)$ are their corresponding estimated patient-level percentages (means of patient’ slices percentages). For Equation (9), $\bar{y_{s_{i}}}$ and $\bar{\hat{y_{s_{i}}}}$ are the means of the ground-truth patient percentages and the estimated ones, respectively.

MAE and RMSE are error indicators where the smaller values indicates better performance. From other hand, PC is a statistic measurement of linear correlation between two variables *Y* and $\hat{Y}$. A value of 1 means that there is a total positive linear correlation and 0 indicates no linear correlation.

## 4. Results

To train and test the CNN architectures (ResneXt-50, DenseNet-161, and Inception-v3), we used the Pytorch [28] library, and a SGD optimizer with momentum equal to 0.9 is used during the training phase. All experiments were carried out on PC with 64 GB Ram and NVIDIA GPU Device Geforce TITAN RTX 24 GB (National Research Council (CNR-ISASI) of Italy, 73100 Lecce, Italy). Each CNN architecture was trained for 30 epochs with initial learning rate of $10^{-4}$ with decays by 0.1 every 10 epochs and batch size equals 20. In addition, we used two active data augmentation techniques: we used random crop data augmentation followed by random rotation using an angle between −10 to 10 degrees. In summary, our experiments are divided into two scenarios: In the first scenario, we used retrained models of ImageNet, while in the second scenario, we used pretrained models that were trained on medical imaging task.

### 4.1. First Scenario

**Figure 5:**
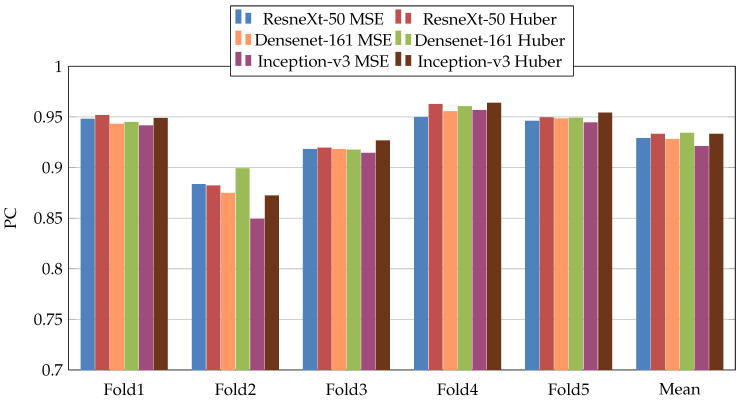
PearsonCorrelation coefficient (PC) results of the first scenario.

**Figure 6:**
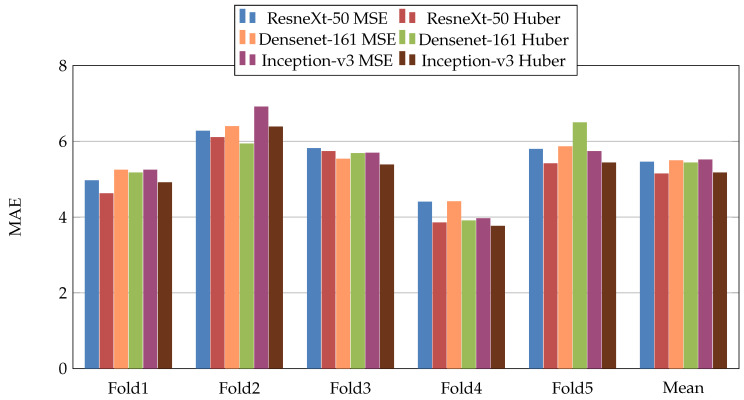
Mean Absolute Error (MAE), results of the first scenario.

**Figure 7:**
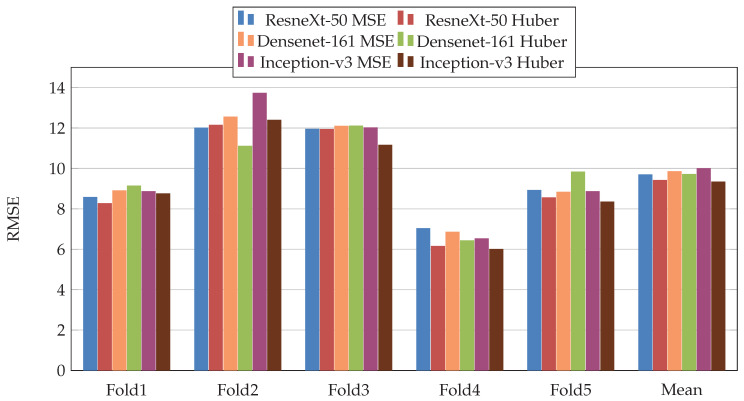
Root Mean Square Error (RMSE), results of the first scenario.

**Figure 8:**
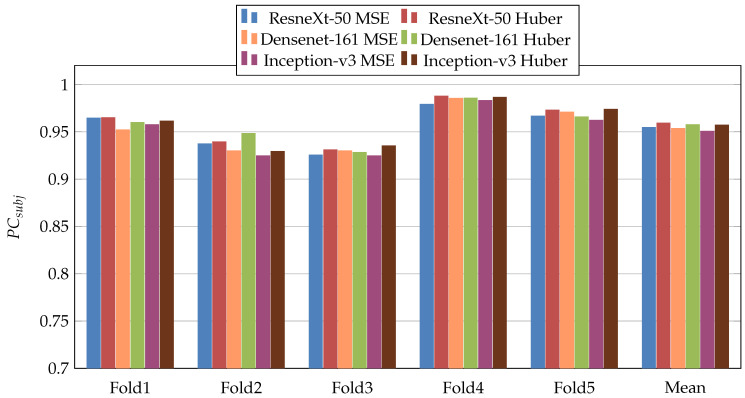
$PC_{subj}$ results of the first scenario.

**Figure 9:**
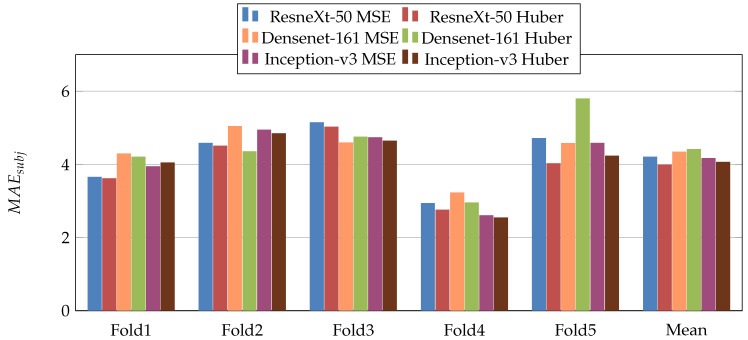
$MAE_{subj}$ results of the first scenario.

**Figure 10:**
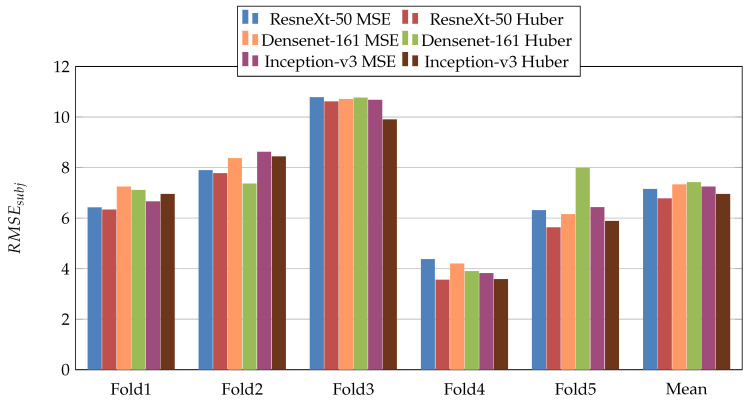
$RMSE_{subj}$ results of the first scenario.

In the first scenario, we used three pretrained CNN architectures on ImageNet [29] (ResneXt-50 [30], Inception-V3 [31], and Densenet-161 [32]). Moreover, we used two loss functions: MSE and Dynamic Huber. Figure 5, Figure 6, Figure 7, Figure 8, Figure 9 and Figure 10 summarize the obtained results of the first scenario for PC, MAE, RMSE, $PC_{subj}$, $MAE_{subj}$, and $RMSE_{subj}$, respectively. From the results, we notice that for all models almost the Dynamic Huber loss gives better results then MSE loss function. This proves the efficiency of using Dynamic Huber loss function for this regression task. On the other hand, we notice that the three trained models with Huber dynamic loss achieved close results. In details, ResneXt-50 achieved the best results performance in MAE, $PC_{subj}$, $MAE_{subj}$, and $RMSE_{subj}$, while Densenet-161 achieved the best result for PC metric and Inception-v3 for RMSE metric. In addition, we notice that Folds 2 and 3 are more challenging compared with Folds 1, 4, and 5, this is probably because these two folds contain more challenging patients than the other folds.

**Figure 11:**
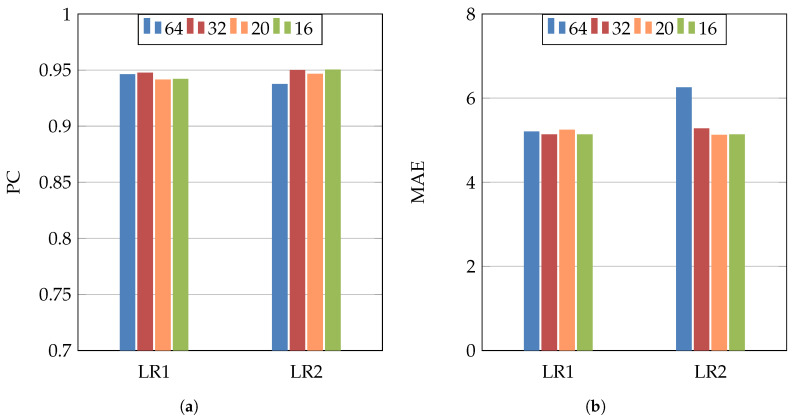
(**a**) PC, (**b**) MAE. Inception-v3 architecture and MSE loss function for studying the influence of two learning rates (LR1 = $10^{-4}$ and LR2 = $10^{-3}$) and four batch sizes (64, 32, 20, and 16).

In order to study the influence of changing the hyperparameters, we used Inception-v3 architecture, MSE loss function, different values of the batch size (64, 32, 20, and 16), and two different initial learning rates ($10^{-4}$ and $10^{-3}$). Figure 11 shows the PC and MAE results of these experiments. From these results, we notice that changing the learning rate or the batch size has no big influence on the results. On the other hand, using smaller batch size (32–16) gave slightly better performance, as the dataset has a medium size.

### 4.2. Second Scenario

In the second scenario, we use the same models as the first scenario but this time they were trained on the recognition of COVID-19 from X-ray scans [2]. In more detail, four lung diseases plus neutral were used to train the CNN architectures [2]. The objective of this scenario is to study the influence of the pretrained model which was trained on medical imaging task. The experimental results are summarized in Table 4. From these results, we notice that Inception-v3 achieved the best performance. Similar to the results of the first scenario, the Dynamic Huber loss gives better results than MSE loss function for most of the evaluation metrics.

**Figure 12:**
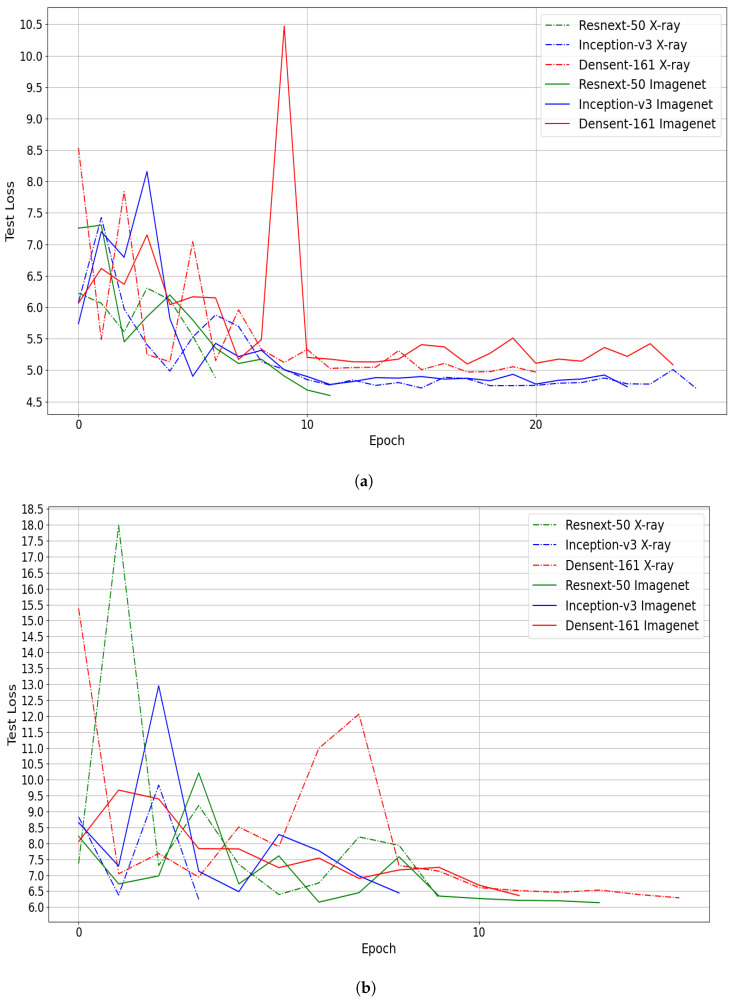
(**a**) Fold1 and (**b**) Fold2. Convergence comparison: X-ray pretrained models vs. ImageNet pretrained models using Huber loss function.

As the pretrained models using X-ray data showed performance improvement compared with ImageNet pretrained models, we investigate the converging speed of each training scenario. To this end, we compare the convergence of the three CNN architectures using Huber loss function and Fold 1 and 2 splits. From Figure 12, we notice that the pretrained models on X-ray data converge faster to the best performance than the ImageNet pretrained models in four out of six experiments. Consequently, using pretrained model trained on medical imaging not only improve the performance, but it can speed up the training process.

## 5. Discussion

The comparison between the first and second scenario experiments shows that the pretrained models on medical imaging task give better result than the pretrained models of ImageNet. From all experiments, we conclude that the best scenario for COVID-19 infection percentage estimation is by using Inception-v3 architecture with an X-ray pretrained model and Dynamic Huber loss function.

To calculate the required time to estimate the COVID-19 infection percentage, we used a PC with Intel i7-CPU (3.60 GHz × 8), 64 GB Ram, and NVIDIA GPU Device Geforce TITAN RTX 24 GB. Table 5 shows the testing time of the three CNN architectures. In the second column, we calculated the required time for one slice. As the number of slices is different from one patient to another, we calculated the required time of batch size of 120 slices (third column of Table 5). The goal of calculating this time is to estimate the required time for volumetric CT-scan, where we calculate the testing time of 120 slices. From Table 5, we notice that the testing time is close for all CNN architectures with slightly lower required testing time for ResneXt-50 architecture for both 1 and 120 slices. Moreover, the required testing time of one volumetric CT-scan is very small (~0.2 s). This proves that estimating the COVID-19 infection percentage using CNN architectures can be applied as real-time applications.

## 6. Conclusions

In this paper, we introduced the Per-COVID-19 dataset, which presents COVID-19 infection percentage estimation from CT scans. The estimation of COVID-19 infection percentage can help quantify the infection and monitor the evolution of the disease. In addition, the required time and efforts from the radiologists to estimate the COVID-19 infection are less compared with infection segmentation labeling. This can help in constructing a large dataset for COVID-19 severity tracking with reasonable time.

Moreover, we evaluated the performance of three CNN architectures: ResneXt-50, Densenet-161, and Inception-v3. For the three CNN architectures, we used two loss functions which are MSE and Dynamic Huber loss. In addition, we evaluate two pretrained models scenarios. In the first scenario, we used ImageNet pretrained models. In the second scenario, we used pretrained models that where trained on X-ray scans to investigate the influence of using pretrained models that were trained on medical imaging task.

The experimental results show that using the X-ray pretrained models improve the results. Moreover, the experiments using Dynamic Huber loss function achieved better performance than the ones used standard MSE loss function. From other hand, Inception-v3 outperformed ResneXt-50 and Densenet-161 architectures in both scenarios. The required time to estimate the COVID-19 infection from a slice is ~0.02 (s). On the other hand, the required time for a CT scan of 120 slices is approximately 0.2 s, which is very small compared with expert radiologists. Both results and testing time prove that is possible to implement real-time application for COVID-19 infection percentage estimation.

Despite the fact that the constructed database consists of 183 CT scans and 3986 slices, adding more CT scans and slices will provide more generalization ability to estimate the COVID-19 infection percentage. From the COVID-19 percentage slices histogram, it is clear that the dataset has less severe slices than other slices categories. This makes estimating the COVID-19 infection percentage for the sever cases more challenging than the other cases. As future future work, we propose to test other CNN architectures with using different data augmentation techniques. Including more labeled CT scans from different devices with different recording settings will help improve the results.

## Figures and Tables

**Table 1:** COVID-19 recognition methods with pros and cons for each method.

COVID-19 Recognition Method	Pros	Cons
RT-PCR	The most used method	Requires specific equipment, Time-consuming, Expensive, Considerable False-Negative Rate
X-ray scans	Low cost, Available in most Hospitals, fast acquisition time	Require an experienced Radiologist
CT scans	Available in most Hospitals, Very accurate recognition, Can be used for monitoring the evolution of the infection, Useful for Infection Severity Prediction	Require an experienced Radiologist, Expensive, Medium acquisition time

**Table 2:** COVID-19 segmentation databases.

Database	# CT-Scan	# Slices
COVID-19 CT segmentation dataset [24] March 2020	40 CT-scans	100 slices
Segmentation dataset nr. 2 [24] April 2020	9 CT-scans	Total of 829 slices from which 373 infected slices
MOSMEDDATA Segmentation [25] May 2020	50 (the 10th slice was kept from each CT-scan)	50
COVID-19-CT-Seg-Benchmark dataset [26] April 2021	20 COVID-19 CT-scans	Total: 1800 slices with 300 infected slices
The proposed dataset	183 CT-scans	3986 labelled slices, where COVID-19 Infection percentage were estimated by two expert Radiologists

**Table 3:** Per-COVID-19 dataset construction.

Hospital	Hakim Saidane Biskra	Ziouch Mohamed Tolga
**Number of CT-scans**	150	33
**Device Scanner**	Hitachi ECLOS CT-Scanner	Toshiba Alexion CT-Scanner
**Slice Thickness**	5 mm	3 mm

**Table 4:** Fivefold cross-validation results of the second scenario (pretrained X-ray models) using three CNN architectures—ResneXt-50, Densenet-161, and Inception-v3—and two loss functions (MSE, Huber). ^§^ Mean and STD were calculated using the average and the standard deviation of the five folds results, respectively. ^*^ the evaluation metrics were calculated using all five folds predictions. The bold is for the results of the five folds. The red, purple and blue colors are for the best performances for Mean (5 folds), STD (5 folds) and All Predictions, respectively.

Architecture	Fold	PC ↑	MAE ↓	RMSE ↓	${PC}_{subj}$↑	${MAE}_{subj}$↓	${RMSE}_{subj}$↓
	Fold 1	0.9453	5.24	8.77	0.9619	4.05	6.58
	Fold 2	0.8569	6.68	13.39	0.9234	4.87	8.70
ResneXt-50	Fold 3	0.9083	6.17	12.53	0.9196	5.23	11.17
MSE	Fold 4	0.9589	3.97	6.32	0.9884	2.31	3.22
	Fold 5	0.9457	5.99	9.05	0.9674	4.92	6.43
	**Mean (5 folds)** ^§^	**0.9230**	**5.61**	**10.01**	**0.9521**	**4.28**	**7.22**
	**STD (5 folds)** ^§^	**0.0371**	**0.9412**	**2.6019**	**0.0265**	**1.0577**	**2.6391**
	**All Predictions** ^*^	**0.9173**	**5.62**	**10.41**	**0.9431**	**4.29**	**7.91**
	Fold 1	0.9487	4.96	8.48	0.9633	3.80	6.43
	Fold 2	0.8227	6.93	15.29	0.9147	4.96	9.25
ResneXt-50	Fold 3	0.9191	5.35	11.84	0.9301	4.27	10.47
Huber	Fold 4	0.9622	3.70	6.09	0.9873	2.39	3.26
	Fold 5	0.9507	5.50	8.80	0.9703	4.34	6.30
	**Mean (5 folds)** ^§^	**0.9207**	**5.29**	**10.10**	**0.9532**	**3.95**	**7.14**
	**STD (5 folds)** ^§^	**0.0510**	**1.0367**	**3.1735**	**0.0267**	**0.8637**	**2.5221**
	**All Predictions** ^*^	**0.9132**	**5.30**	**10.65**	**0.9427**	**4.02**	**7.87**
	Fold 1	0.9438	5.15	8.65	0.9611	3.79	6.36
	Fold 2	0.8768	6.48	12.34	0.9354	4.62	8.04
Densenet-161	Fold 3	0.9116	5.86	12.44	0.9259	4.73	10.89
MSE	Fold 4	0.9576	4.11	6.65	0.9861	2.97	4.14
	Fold 5	0.9479	5.79	8.99	0.9691	4.82	6.38
	**Mean (5 folds)** ^§^	**0.9275**	**5.48**	**9.81**	**0.9555**	**4.18**	**7.16**
	**STD (5 folds)** ^§^	**0.0297**	**0.8032**	**2.2503**	**0.0220**	**0.7101**	**2.2385**
	**All Predictions** ^*^	**0.9234**	**5.49**	**10.12**	**0.9501**	**4.09**	**7.56**
	Fold 1	0.9440	4.97	8.78	0.9569	3.91	6.84
	Fold 2	0.8938	6.14	11.36	0.9459	4.57	7.58
Densenet-161	Fold 3	0.9158	5.89	12.33	0.9290	4.99	10.87
Huber	Fold 4	0.9643	3.67	5.93	0.9893	2.33	3.16
	Fold 5	0.9526	5.48	8.71	0.9697	4.56	6.54
	**Mean (5 folds)** ^§^	**0.9341**	**5.23**	**9.42**	**0.9582**	**4.07**	**7.00**
	**STD (5 folds)** ^§^	**0.0257**	**0.8749**	**2.2505**	**0.0205**	**0.9369**	**2.4615**
	**All Predictions** ^*^	**0.9305**	**5.25**	**9.75**	**0.9523**	**4.06**	**7.51**
	Fold 1	0.9483	5.22	8.51	0.9630	3.95	6.53
	Fold 2	0.8571	6.63	13.14	0.9258	5.01	8.57
Inception-v3	Fold 3	0.9111	5.89	12.28	0.9240	5.22	10.84
MSE	Fold 4	0.9605	3.91	6.22	0.9878	2.52	3.27
	Fold 5	0.9463	6.10	9.22	0.9667	5.02	6.74
	**Mean (5 folds)** ^§^	**0.9247**	**5.55**	**9.87**	**0.9535**	**4.34**	**7.19**
	**STD (5 folds)** ^§^	**0.0375**	**0.9362**	**2.5335**	**0.0248**	**1.0149**	**2.4992**
	**All predictions**	**0.9163**	**5.90**	**10.57**	**0.9470**	**4.16**	**7.51**
	Fold 1	0.9526	4.81	8.43	0.9673	3.81	6.45
	Fold 2	0.8905	6.17	11.64	0.9414	4.59	7.63
Inception-v3	Fold 3	0.9230	5.47	11.69	0.9324	4.75	10.41
Huber	Fold 4	0.9607	3.79	6.33	0.9850	2.85	3.91
	Fold 5	0.9556	5.29	8.14	0.9753	4.04	5.56
	**Mean (5 folds)** ^§^	**0.9365**	**5.10**	**9.25**	**0.9603**	**4.01**	**6.79**
	**STD (5 folds)** ^§^	**0.0264**	**0.7896**	**2.1022**	**0.0201**	**0.6738**	**2.1786**
	**All Predictions** ^*^	**0.9330**	**5.34**	**9.44**	**0.9558**	**3.98**	**6.87**

**Table 5:** Average of five testing times for the evaluated CNN architectures (ResneXt-50, Densenet-161 and Inception-v3).

Model	Testing Time of One Slice (s)	Testing Time of 120 Slices (s)
ResneXt-50	0.012470	0.171227
DenseNet-161	0.023900	0.205754
Inception-v3	0.013844	0.185773

## Data Availability

The data were collected from the Hospitals of Hakim Saidane Biskra and Ziouch Mohamed Tolga (Algeria) and it was made available at https://github.com/faresbougourzi/Per-COVID-19 (Last accessed on 4 August 2021).

## Abbreviations

The following abbreviations are used in this manuscript:

CNN	Convolutional Neural Network
RT-PCR	Reverse Transcription Polymerase Chain Reaction
